# Asymmetric Power Hazard Distribution for COVID-19 Mortality Rate under Adaptive Type-II Progressive Censoring: Theory and Inferences

**DOI:** 10.1155/2022/5134507

**Published:** 2022-04-20

**Authors:** Mahmoud El-Morshedy, Rashad M. El-Sagheer, Mohamed S. Eliwa, Khaled M. Alqahtani

**Affiliations:** ^1^Department of Mathematics, College of Science and Humanities in Al-Kharj, Prince Sattam Bin Abdulaziz University, Al-Kharj 11942, Saudi Arabia; ^2^Department of Statistics and Computer Science, Faculty of Science, Mansoura University, Mansoura 35516, Egypt; ^3^Mathematics Department, Faculty of Science, Al-Azhar University, Naser City 11884, Cairo, Egypt; ^4^Department of Statistics and Operation Research, College of Science, Qassim University, P.O. Box 6644, Buraydah 51482, Saudi Arabia; ^5^Department of Mathematics, Faculty of Science, Mansoura University, Mansoura 35516, Egypt

## Abstract

This article investigates the estimation of the parameters for power hazard function distribution and some lifetime indices such as reliability function, hazard rate function, and coefficient of variation based on adaptive Type-II progressive censoring. From the perspective of frequentism, we derive the point estimations through the method of maximum likelihood estimation. Besides, delta method is implemented to construct the variances of the reliability characteristics. Markov chain Monte Carlo techniques are proposed to construct the Bayes estimates. To this end, the results of the Bayes estimates are obtained under squared error and linear exponential loss functions. Also, the corresponding credible intervals are constructed. A simulation study is utilized to assay the performance of the proposed methods. Finally, a real data set of COVID-19 mortality rate is analyzed to validate the introduced inference methods.

## 1. Introduction

To achieve a balance between the total time spent in the experiment and the number of units used in the experiment, the experiment must be subjected to a good control system (censoring scheme) that allows the experimenter to obtain results that enable him to make good statistical inference. At the same time, the working experimental units are saved for future use, as well as the cost and time associated with testing. Type-I (time) and Type-II (failure of units) censoring schemes are the oldest and most common censoring schemes. In these two types of censoring schemes, units cannot be withdrawn from an experiment until the final stage or the number of units fails.

**Figure 1:**
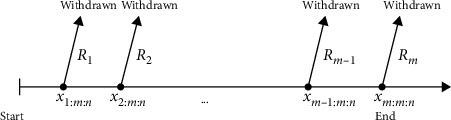
Description of progressive Type-II censoring scheme.

Balakrishnan and Sandhu [1] introduced the progressive Type-II censoring that has more flexibility than the Type-II censoring in allowing units to be withdrawn from the test at different observed failure times. Briefly, it can be described as follows: assume that *n* independent units are placed in the life test and the observed failure times *m*(*m* ≤ *n*), which are called a progressive sample as *X*_1:*m*:*n*_ < *X*_2:*m*:*n*_ < …<*X*_*m*:*m*:*n*_, are prefixed. Besides, the scheme **R**=(*R*_1_, *R*_2_,…, *R*_*m*_) is a prefixed censoring plan. At the first failure occurrence *X*_1_, the surviving units *R*_1_ are randomly withdrawn from the test; then, at the second failure occurrence *X*_2_, the surviving units *R*_2_ are randomly withdrawn from the test and so on until the occurrence of the *m*^th^ failure and therefore the remaining surviving units *R*_*m*_=*n* − *m* − ∑_*i*=1_^*m*−1^*R*_*i*_ are withdrawn from the test and the test is terminated; see Figure 1. For more details and extensive reviews of the literature on progressive Type II censoring, readers may refer to Balakrishnan [2], Balakrishnan and Sandhu [3], and EL-Sagheer [4].

**Figure 2:**
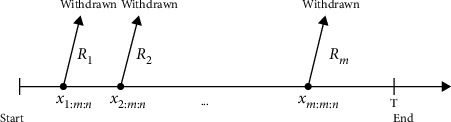
Experiment terminates before time *T*, that is, *X*_*m*:*m*:*n*_ < *T*.

**Figure 3:**
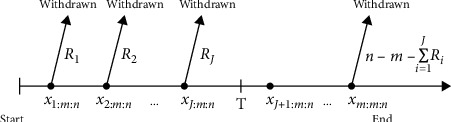
Experiment terminates after time *T*, that is, *X*_*m*:*m*:*n*_ ≥ *T*.

Kundu and Joarder [5] introduced a Type-II progressive hybrid censoring scheme; the experiment in this type is terminated at a prescribed time *T* with a progressive Type-II right censored. It is clear that this type of censoring has the same disadvantage of the Type-I censoring (time), in which *m* is random and may be a small number. For this reason, Ng et al. [6] developed the adaptive Type-II progressive censoring scheme as a mix of Type-II progressive censoring and Type-I censoring. This type allows *R*_1_, *R*_2_,…, *R*_*m*_ to depend on the failure times so that the effective sample size is always *m*, which is fixed in advance. Thus, the life testing experiment can save both the total test time and the cost induced by failure of the units. This censoring scheme can be described as follows: consider *n* identical units under observation in a life testing experiment and suppose the experimenter provides a time *T*, which is an ideal total test time, but we may allow the experiment to run over time *T*. If the *m*^th^ progressively censored observed failure occurs before time *T* (i.e., *X*_*m*:*m*:*n*_ < *T*), the experiment terminates at the time *X*_*m*:*m*:*n*_. Otherwise, once the experimental time passes time *T* but the number of observed failures has not reached *m*, we would want to terminate the experiment as soon as possible. Therefore, we should leave as many surviving units on the test as possible. Suppose *J* is the number of failures observed before time *T*, that is, *X*_*J*:*m*:*n*_ ≤ *T* < *X*_*J*+1:*m*:*n*_, *J*=0,1,…, *m*, where *X*_0:*m*:*n*_ ≡ 0 and *X*_*m*+1:*m*:*n*_ ≡ *∞*. After the experiment passed time *T*, we set *R*_*J*+1_=…=*R*_*m*−1_=0 and *R*_*m*_=*n* − *m* − ∑_*i*=1_^*J*^*R*_*i*_. This formulation leads us to terminate the experiment as soon as possible if the (*J*+1)th failure time is greater than *T* for *J*+1 < *m*. One extreme case is when *T*⟶*∞*, which means time is not the main consideration for the experimenter; then we will have a usual progressive Type-II censoring scheme with the prefixed progressive censoring scheme (*R*_1_,…, *R*_*m*_). Another extreme case can occur when *T*=0, which means we always want to end the experiment as soon as possible; then, we will have *R*_1_,…, *R*_*m*−1_=0 and *R*_*m*_=*n* − *m*, which results in the conventional Type-II censoring scheme. For extensive reviews of the literature on the adaptive Type-II progressive censoring scheme, readers may refer to Cramer and Iliopoulos [7], Mohie El-Din et al. [8], and El-Sagheer et al. [9], which can be briefly described by Figures 2 and 3.

From Figures 1 and 2, we observe that the value of *T* plays an important role in the determination of the values of *R*_*i*_ , *i*=1,2,…, *m*, and is a compromise between a shorter experimental time and a higher chance to observe extreme failures.

Mugdadi [10] proposed the two-parameter power hazard function distribution, denoted by PHFD (*θ*, *λ*) as an alternative to the Weibull, Rayleigh, and exponential distributions and studied its different properties. This distribution has increasing hazard rate function (HRF) for *λ* > 0, and it has decreasing HRF for −1 < *λ* < 0. The probability density function (PDF), cumulative distribution function (CDF), reliability function RF, and HRF are given, respectively, by

(1) $$\begin{array}{c} f\left(x;\theta ,\lambda \right)=\theta x^{\lambda }\exp\left\{-\frac{\theta }{\lambda +1}x^{\lambda +1}\right\},\theta >0,\lambda >-1,x>0, \end{array}$$

(2) $$\begin{array}{c} r\left(x\right)=\exp\left\{-\frac{\theta }{\lambda +1}x^{\lambda +1}\right\},\theta >0,\lambda >-1,x>0, \end{array}$$

(3) $$\begin{array}{c} h\left(x\right)=\theta x^{\lambda },\theta >0,\lambda >-1,x>0, \end{array}$$

where *θ* and *λ* are the scale and shape parameters, respectively. The PHFD has been shown to be useful for modeling and analyzing the life time data in medical and biological sciences, engineering, and so on. This distribution is a very flexible model that approaches different models when its parameters are changed. It contains the following special models: PHFD refers to Rayleigh (*β*) when *θ*=1/*β*^2^ and *λ*=1, PHFD reduces to Weibull (*θ*, 1) when *λ*=*θ* − 1, and PHFD is exponential distribution with mean 1/*θ* when *λ*=0.

The coefficient of variation is used in many science areas such as medical sciences, chemistry, biology, economics, finance, engineering, and reliability theory to assess homogeneity of bone test samples, dose-response studies, wildlife studies, and other fields; see Subrahmanya Nairy and Rao [11], Creticos et al. [12], Versaci et al. [13], and El-Sagheer et al. [9]. Given a set of observations from PHFD (*θ*, *λ*), the sample coefficient of variation (*CV*) is

(4) $$\begin{array}{c} CV=\frac{\sqrt{E\left(X^{2}\right)-{\left[E\left(X\right)\right]}^{2}}}{E\left(X\right)},E\left(X\right)\neq 0, \end{array}$$

where *E*(*X*) and *E*(*X*^2^) are the first and the second moments of the PHFD (*θ*, *λ*), given by

(5) $$\begin{array}{c} E\left(X\right)={\left(\frac{\theta }{\lambda +1}\right)}^{-1/\lambda +1}\Gamma \left(\frac{\lambda +2}{\lambda +1}\right), \end{array}$$

(6) $$\begin{array}{c} E\left(X^{2}\right)={\left(\frac{\theta }{\lambda +1}\right)}^{-2/\lambda +1}\Gamma \left(\frac{\lambda +3}{\lambda +1}\right), \end{array}$$

where Γ(*ϖ*) is the gamma function Γ(*ϖ*)=∫_0_^*∞*^*z*^*ϖ*−1^*e*^−*z*^*dz*. Thus,

(7) $$\begin{array}{c} CV=Q\left(\lambda \right), \end{array}$$

where

(8) $$\begin{array}{c} Q\left(\lambda \right)=\frac{\sqrt{\Gamma \left(\lambda +3/\lambda +1\right)-{\left[\Gamma \left(\lambda +2/\lambda +1\right)\right]}^{2}}}{\Gamma \left(\lambda +2/\lambda +1\right)},\lambda >\text{0}. \end{array}$$

Inferences for the PHFD have been studied by several authors including Kınacı [14] discussed the stress-strength reliability model for PHFD, El-Sagheer [15] investigated the problem of point and interval estimations of the parameters for PHFD based on progressive Type-II censoring scheme, Mugdadi and Min [16] discussed Bayes estimation of PHFD based on a complete and Type-II censored samples, and Khan [17] established the recurrence relations for single and product moments of generalized order statistics from the PHFD. Recently, El-Sagheer et al. [18] constructed the Bayesian and non-Bayesian approaches for the lifetime performance index with the progressive Type-II censored sample from the PHFD. In this article, we use the adaptive Type-II progressive censored (AT2PC) scheme to study the problem of estimating the shape and scale parameters, RF, HRF, and*CV* for two-parameter PHFD.

The structure of the paper is organized as follows.  Section 2 deals with the maximum likelihood estimate and asymptotic confidence intervals. Bayesian estimates using Markov chain Monte Carlo technique are provided in Section 3. In Section 4, a simulation study is conducted to compare the performance of these estimation methods. A real data set of COVID-19 is presented to illustrate the application of the proposed inferences in Section 5. Finally, a brief conclusion is given in Section 6.

## 2. Maximum Likelihood Inference

Suppose that *x*=*X*_1:*m*:*n*_^*R*^<*X*_2:*m*:*n*_^*R*^ < …<*X*_*m*:*m*:*n*_^*R*^ is a AT2PC-order statistics from the PHFD with progressive censored scheme *R*=(*R*_1_, *R*_2_,…, *R*_*m*_). According to Ng et al. [6], the log-likelihood function $\ell =\log\;L\left(\theta ,\lambda ;\underline{x}\right)$ without normalized constant can be written as

(9) $$\begin{array}{c} \ell \propto m\;\log\;\theta +\lambda \overset{m}{\underset{i=1}{\sum }}\log\;x_{i}-\frac{\theta }{\lambda +1}\left[\overset{m}{\underset{i=1}{\sum }}R_{i}x_{i}^{\lambda +1}+\overset{j}{\underset{i=1}{\sum }}x_{i}^{\lambda +1}+\left(n-m-\sum_{i=1}^{J}R_{i}\right)x_{m}^{\lambda +1}\right], \end{array}$$

where *x*_*i*_ is used instead of *x*_*i*:*m*:*n*:*k*_^*R*^. After differentiating *ℓ* with respect to *θ* and *λ*, respectively, and equating each of them to zero, the likelihood equations can be written as follows:

(10) $$\begin{array}{c} \frac{m}{\theta }-\frac{1}{\lambda +1}\left[\sum_{i=1}^{m}R_{i}x_{i}^{\lambda +1}+\sum_{i=1}^{J}x_{i}^{\lambda +1}+\left(n-m-\sum_{i=1}^{J}R_{i}\right)x_{m}^{\lambda +1}\right]=0, \end{array}$$

(11) $$\begin{array}{c} \overset{m}{\underset{i=1}{\sum }}\log\;x_{i}-\frac{\theta }{\lambda +1}\left[\overset{m}{\underset{i=1}{\sum }}R_{i}x_{i}^{\lambda +1}\log\;x_{i}+\overset{j}{\underset{i=1}{\sum }}x_{i}^{\lambda +1}\log\;x_{i}+\left(n-m-\sum_{i=1}^{J}R_{i}\right)x_{m}^{\lambda +1}\log\;x_{m}\right]+\frac{\theta }{{\left(\lambda +1\right)}^{2}}\left[\sum_{i=1}^{m}R_{i}x_{i}^{\lambda +1}+\sum_{i=1}^{J}x_{i}^{\lambda +1}+\left(n-m-\sum_{i=1}^{J}R_{i}\right)x_{m}^{\lambda +1}\right]=0. \end{array}$$

From (10), the MLE of *θ* can be illustrated as

(12) $$\begin{array}{c} \hat{\theta }=m{\left[\frac{1}{\hat{\lambda }+1}\left\{\sum_{i=1}^{m}R_{i}x_{i}^{\hat{\lambda }+1}+\sum_{i=1}^{J}x_{i}^{\hat{\lambda }+1}+\left(n-m-\sum_{i=1}^{J}R_{i}\right)x_{m}^{\hat{\lambda }+1}\right\}\right]}^{-1}. \end{array}$$

Since (10) and (12) do not have closed-form solutions, Newton–Raphson iteration method is widely used to obtain the desired MLEs in such situations. For more details, see El-Sagheer [4]. Once MLEs of *θ* and *λ* are obtained, the MLEs of *r*(*t*), *h*(*t*), and *CV* for a given mission time *t* can be obtained after replacing *θ* and *λ* by $\hat{\theta }$ and $\hat{\lambda }$ according to the invariant property of the MLEs as

(13) $$\begin{array}{c} \hat{r}\left(t\right)=\exp\left\{-\frac{\hat{\theta }}{\hat{\lambda }+1}t^{\hat{\lambda }+1}\right\},\\ \hat{h}\left(t\right)=\hat{\theta }t^{\hat{\lambda }},\\ \hat{C}V=Q\left(\hat{\lambda }\right). \end{array}$$

### 2.1. Approximate Confidence Intervals

According to Cohen [19], the Fisher information matrix (FIM), which is defined by the negative expectation of the partial second derivative of the log-likelihood function, is needed to construct the approximate confidence intervals (ACIs) for parameters Λ=(*θ*, *λ*).

(14) $$\begin{array}{c} I\left(\Lambda \right)=E{\left[-\frac{\partial^{2}\ell }{\partial \Lambda_{i}\partial \Lambda_{l}}\right]}_{i,l=1,2}\\ ={\left[I_{il}\right]}_{2\times 2}. \end{array}$$

The elements of the FIM are

(15) $$\begin{array}{c} I_{11}=\frac{-m}{\theta^{2}},\\ I_{21}=-\frac{1}{\lambda +1}\left[\sum_{i=1}^{m}R_{i}x_{i}^{\lambda +1}\log\;x_{i}+\sum_{i=1}^{J}x_{i}^{\lambda +1}\log\;x_{i}+\left(n-m-\sum_{i=1}^{J}R_{i}\right)x_{m}^{\lambda +1}\log\;x_{m}\right]+\frac{1}{{\left(\lambda +1\right)}^{2}}\left[\sum_{i=1}^{m}R_{i}x_{i}^{\lambda +1}+\sum_{i=1}^{J}x_{i}^{\lambda +1}+\left(n-m-\sum_{i=1}^{J}R_{i}\right)x_{m}^{\lambda +1}\right], \end{array}$$

(16) $$\begin{array}{c} I_{22}=-\frac{\theta }{\lambda +1}\left[\sum_{i=1}^{m}R_{i}x_{i}^{\lambda +1}{\left(\log\;x_{i}\right)}^{2}+\sum_{i=1}^{J}x_{i}^{\lambda +1}{\left(\log\;x_{i}\right)}^{2}+\left(n-m-\sum_{i=1}^{J}R_{i}\right)x_{m}^{\lambda +1}{\left(\log\;x_{m}\right)}^{2}\right]+\frac{2\theta }{{\left(\lambda +1\right)}^{2}}\left[\sum_{i=1}^{m}R_{i}x_{i}^{\lambda +1}\log\;x_{i}+\sum_{i=1}^{J}x_{i}^{\lambda +1}\log\;x_{i}+\left(n-m-\sum_{i=1}^{J}R_{i}\right)x_{m}^{\lambda +1}\log\;x_{m}\right]-\frac{2\theta }{{\left(\lambda +1\right)}^{3}}\left[\sum_{i=1}^{m}R_{i}x_{i}^{\lambda +1}+\sum_{i=1}^{J}x_{i}^{\lambda +1}+\left(n-m-\sum_{i=1}^{J}R_{i}\right)x_{m}^{\lambda +1}\right]. \end{array}$$

Since MLE has asymptotic normality property under certain regularity conditions, the estimator $\hat{\Lambda }=\left(\hat{\theta },\hat{\lambda }\right)$ has asymptotic distribution $\hat{\Lambda }-\Lambda ⟶N\left(0,I^{-1}\left(\Lambda \right)\right)$, and the inverse matrix of *I*(Λ) is

(17) $$\begin{array}{c} I^{-1}\left(\hat{\Lambda }\right)=\left[\begin{array}{cc} \text{Var}\left(\hat{\theta }\right) & \text{Cov}\left(\hat{\theta },\hat{\lambda }\right)\\ \text{Cov}\left(\hat{\lambda },\hat{\theta }\right) & \text{Var}\left(\hat{\lambda }\right) \end{array}\right]. \end{array}$$

Thus, the 100(1 − *γ*)% ACIs of Λ is

(18) $$\begin{array}{c} \left[{\hat{\Lambda }}_{i}-z_{\gamma /2}\sqrt{\text{Var}\left({\hat{\Lambda }}_{i}\right)},{\hat{\Lambda }}_{i}+z_{\gamma /2}\sqrt{\text{Var}\left({\hat{\Lambda }}_{i}\right)}\right],i=1,2, \end{array}$$

where $\text{Var}\left({\hat{\Lambda }}_{i}\right)=I_{ii}^{-1},i=1,2$. *z*_*γ*/2_ is the percentile of the standard normal distribution with right-tail probability *γ*/2.

### 2.2. Delta Method

In order to construct the ACIs of *r*(*t*), *h*(*t*), and CV, we need to find the variances of them. So, Greene [20] implemented the delta method for this purpose. In this method, for analytically computing the variance, a linear approximation of the functions of the MLEs is created, and then the variance of the simpler linear function that can be used for large sample inference is computed. Let *W*=(∂Δ/∂*θ*, ∂Δ/∂*λ*) be the first partial derivative of Δ=(*r*(*t*), *h*(*t*), CV). Then, the approximate estimates of variances $\hat{\Delta }=\left(\hat{r}\left(t\right),\hat{h}\left(t\right),\hat{\text{C}}\text{V}\right)$ can be written as

(19) $$\begin{array}{c} \text{Var}\left(\hat{\Delta }\right)≃{\left(W^{T}I^{-1}\left(\hat{\Lambda }\right)W\right)}_{↓\left(\Lambda =\hat{\Lambda }\right)}, \end{array}$$

where *W*^*T*^ is the transpose matrix of *W*. Thus, the (1 − *γ*)100% ACIs for $\hat{\Delta }$ can be given by

(20) $$\begin{array}{c} \left[\hat{\Delta }-z_{\gamma /2}\sqrt{\text{Var}\left(\hat{\Delta }\right)},\hat{\Delta }+z_{\gamma /2}\sqrt{\text{Var}\left(\hat{\Delta }\right)}\right]. \end{array}$$

Moreover, Meeker and Escobar [21] suggested using the natural approximation for the log-transformed MLE to prevent the negative lower bound of the ACIs. Thus, two-sided (1 − *γ*)100% normal ACIs for $\hat{\Theta }=\hat{\theta }$, $\hat{\lambda }$, $\hat{r}\left(t\right)$, $\hat{h}\left(t\right)$ or $\hat{\text{C}}\text{V}$ are given by

(21) $$\begin{array}{c} \left[\hat{\Theta }\exp\left\{-\frac{z_{\gamma /2}\sqrt{\text{Var}\left(\hat{\Theta }\right)}}{\hat{\Theta }}\right\},\hat{\Theta }\exp\left\{\frac{{\text{z}}_{\gamma /2}\sqrt{\text{Var}\left(\hat{\Theta }\right)}}{\hat{\Theta }}\right\}\right]. \end{array}$$

## 3. Bayesian Inference

To characterize the problems more rationally and reasonably, we must take into consideration both the information from observed sample data and the prior information; this is the main idea of Bayesian inference. In this section, Bayesian inference procedures using Markov chain Monte Carlo (MCMC) technique are proposed to estimate the parameters *θ* and *λ* as well as *r*(*t*), *h*(*t*), and CV under both squared error (SE) and linear exponential (LINEX) loss functions. Also, the corresponding CRIs are constructed under MCMC technique. We consider here that the parameters *θ* and *λ* follow the gamma prior distributions with PDFs

(22) $$\begin{array}{c} \left\{\begin{array}{cc} \pi_{1}\left(\theta \right)\propto \theta^{a_{1}-1}\exp\left\{-b_{1}\theta \right\}, & \theta >\text{0},\\ \pi_{2}\left(\lambda \right)\propto \lambda^{a_{2}-1}\exp\left\{-b_{2}\lambda \right\}, & \lambda >\text{0}, \end{array}\right. \end{array}$$

where *a*_1_, *b*_1_, *a*_2_ and *b*_2_ reflect the knowledge of prior about (*θ*, *λ*) and they are assumed to be known and nonnegative hyperparameters. In addition, the parameters *θ* and *λ* as well as the corresponding priors are considered here to be independent. Further, the joint prior of the parameters *θ* and *λ* can be written as

(23) $$\begin{array}{c} \pi \left(\theta ,\lambda \right)\propto \theta^{a_{1}-1}\lambda^{a_{2}-1}\exp\left\{-b_{1}\theta -b_{2}\lambda \right\}. \end{array}$$

Via Bayes' theorem across using the likelihood function $L\left(\theta ,\lambda ;\underline{x}\right)$ with the joint prior *π*(*θ*, *λ*), the joint posterior density $\pi^{*}\left(\theta ,\lambda |\underline{x}\right)$ of *θ* and *λ* can be obtained as

(24) $$\begin{array}{c} \pi^{*}\left(\theta ,\lambda |\underline{x}\right)=\frac{L\left(\theta ,\lambda ;\underline{x}\right)\times \pi \left(\theta ,\lambda \right)}{\int_{0}^{\infty }\int_{0}^{\infty }L\left(\theta ,\lambda ;\underline{x}\right)\times \pi \left(\theta ,\lambda \right)d\theta d\lambda }\propto \theta^{m+a_{1}-1}\lambda^{a_{2}-1}\left[\overset{m}{\underset{i=1}{\prod }}x_{i}^{\beta }\right]\exp\left\{-b_{1}\theta -b_{2}\lambda \right\}\exp\left\{-\frac{\theta }{\lambda +1}\left[\sum_{i=1}^{m}R_{i}x_{i}^{\lambda +1}+\sum_{i=1}^{J}x_{i}^{\lambda +1}+\left(n-m-\sum_{i=1}^{J}R_{i}\right)x_{m}^{\lambda +1}\right]\right\}. \end{array}$$

Under SE loss function, the Bayesian estimator of any function of *θ* and *λ*, say *φ*(*θ*, *λ*), is given by

(25) $$\begin{array}{c} {\hat{\varphi }}_{BS}\left(\theta ,\lambda \right)=E_{\theta ,\lambda |\underline{x}}\left[\varphi \left(\theta ,\lambda \right)\right]\\ =\frac{\int_{0}^{\infty }\int_{0}^{\infty }\varphi \left(\theta ,\lambda \right)L\left(\theta ,\lambda ;\underline{x}\right)\times \pi \left(\theta ,\lambda \right)}{\int_{0}^{\infty }\int_{0}^{\infty }L\left(\theta ,\lambda ;\underline{x}\right)\times \pi \left(\theta ,\lambda \right)d\theta d\lambda }. \end{array}$$

Under LINEX loss function, we have

(26) $$\begin{array}{c} {\hat{\varphi }}_{BL}\left(\theta ,\lambda \right)=E_{\theta ,\lambda |\underline{x}}\left[e^{-c\varphi \left(\theta ,\lambda \right)}\right]\\ =\frac{\int_{0}^{\infty }\int_{0}^{\infty }e^{-c\varphi \left(\theta ,\lambda \right)}L\left(\theta ,\lambda ;\underline{x}\right)\times \pi \left(\theta ,\lambda \right)}{\int_{0}^{\infty }\int_{0}^{\infty }L\left(\theta ,\lambda ;\underline{x}\right)\times \pi \left(\theta ,\lambda \right)d\theta \;d\lambda }, \end{array}$$

where *c* ≠ 0. It is clear that the ratio of two integrals in (25) and (26) cannot be obtained in a closed form. Thus, it is necessary to use suitable numerical methods to approximate these integrals. So, in this case, we apply MCMC technique to obtain the Bayes estimates of *θ*, *λ*, *r*(*t*), *h*(*t*), and CV and corresponding credible intervals (CRIs).

### 3.1. Markov Chain Monte Carlo Technique

MCMC technique is one of the most general technique for an estimation, which is provided here to compute the Bayes estimates and the corresponding CRIs for *θ*, *λ*, *r*(*t*), *h*(*t*), and CV. It is known that there are several procedures of MCMC technique available in which samples are generated from the conditional posterior densities. One of the simplest MCMC procedures is the Gibbs sampling procedure, which was proposed by Geman and Geman [22]. Another procedure is considered the Metropolis–Hastings (M-H) algorithm, which was proposed by Metropolis et al. [23] and later extended by Hastings [24]. A more general procedure of MCMC procedures which we will use is considered the M-H within Gibbs sampling. Gibbs sampler is required to decompose the joint posterior distribution into full conditional distributions for each parameter and then sample from them. From (26), the posterior conditional density function of *θ* given *λ* can be written as

(27) $$\begin{array}{c} \pi_{1}^{*}\left(\theta |\lambda ,\underline{x}\right)\propto \theta^{m+a_{1}-1}\exp\left\{\frac{-\theta }{\lambda +1}\left[\sum_{i=1}^{m}R_{i}x_{i}^{\lambda +1}+\sum_{i=1}^{J}x_{i}^{\lambda +1}+\left(n-m-\sum_{i=1}^{J}R_{i}\right)x_{m}^{\lambda +1}\right]-b_{1}\theta \right\}. \end{array}$$

Similarly,

(28) $$\begin{array}{c} \pi_{2}^{*}\left(\lambda |\theta ,\underline{x}\right)\propto \lambda^{a_{2}-1}\exp\left\{-b_{2}\lambda \right\}\left[\prod_{i=1}^{m}x_{i}^{\beta }\right]\exp\left\{-\frac{\theta }{\lambda +1}\left[\sum_{i=1}^{m}R_{i}x_{i}^{\lambda +1}+\sum_{i=1}^{J}x_{i}^{\lambda +1}+\left(n-m-\sum_{i=1}^{J}R_{i}\right)x_{m}^{\lambda +1}\right]\right\}. \end{array}$$

Moreover, the conditional posterior density of *θ* given in (27) is gamma density with shape parameter (*m*+*a*_1_) and scale parameter (1/*λ*+1[∑_*i*=1_^*m*^*R*_*i*_*x*_*i*_^*λ*+1^+∑_*i*=1_^*J*^*x*_*i*_^*λ*+1^+(*n* − *m* − ∑_*i*=1_^*J*^*R*_*i*_)*x*_*m*_^*λ*+1^] − *b*_1_). Thus, by implementing any gamma generating routine, samples of *θ* can be simply generated. In addition, the conditional posterior density of *λ* cannot be reduced analytically to well-known distribution. So, according to Tierney [25], M-H algorithm within Gibbs sampling with normal proposal distribution is used to conduct the MCMC methodology. The hybrid M-H algorithm and Gibbs sampler works as follows:

(1) Start with an $\left(\theta^{\left(0\right)}=\hat{\theta },\lambda^{\left(0\right)}=\hat{\lambda }\right)$, and set *k*=1.
(2) Generate *θ*^(*k*)^ from gamma distribution $\pi_{1}^{*}\left(\theta |\lambda^{\left(k-1\right)},\underline{x}\right)$.
(3) Using M-H algorithm, generate *λ*^(*k*)^ from $\pi_{2}^{*}\left(\lambda^{\left(k-1\right)}|\theta^{\left(k\right)},\underline{x}\right)$ with the $N\left(\lambda^{\left(j-1\right)},\text{Var}\left(\hat{\lambda }\right)\right)$ proposal distribution, where $\text{Var}\left(\hat{\lambda }\right)$ is from a variance-covariance matrix.
  (a) Generate *λ*^*∗*^ from $N\left(\lambda^{\left(j-1\right)},\text{Var}\left(\hat{\lambda }\right)\right)$.
  (b) Evaluate the acceptance probability
    (29) $$\begin{array}{c} \psi_{\lambda }=\min\left[1,\frac{\pi_{2}^{*}\left(\lambda^{*}|\theta^{\left(k\right)},\underline{x}\right)}{\pi_{2}^{*}\left(\lambda^{\left(j-1\right)}|\theta^{\left(k\right)},\underline{x}\right)}\right]. \end{array}$$
  (c) Generate a *ρ*_1_ from a uniform (0,1) distribution.
  (d) If *ρ*_1_ < *ψ*_*λ*_, accept the proposal and set *λ*^(*k*)^=*λ*^*∗*^; else, set *λ*^(*k*)^=*λ*^(*k* − 1)^.
(4) Compute *r*(*t*), *h*(*t*), and *CV* as
  (30) $$\begin{array}{c} r^{\left(k\right)}\left(t\right)=\exp\left\{-\frac{\theta^{\left(k\right)}}{\lambda^{\left(k\right)}+1}t^{\lambda^{\left(k\right)}+1}\right\}\text{,}\\ {\text{h}}^{\left(\text{k}\right)}\left(\text{t}\right)=\theta^{\left(\text{k}\right)}{\text{t}}^{\lambda^{\left(\text{k}\right)}}\text{,}\\ {\text{CV}}^{\left(\text{k}\right)}=\text{Q}\left(\lambda^{\left(\text{k}\right)}\right). \end{array}$$
(5) Set *k*=*k*+1.
(6) Reiterate Steps (3–5) *N* times.
(7) Under SE and LINEX loss functions, the Bayes estimate of *ζ* (where *ζ*= *α*, *β*, *δ*, *λ*, *r*(*t*), *h*(*t*), and CV) can be obtained by
  (31) $$\begin{array}{c} {\hat{\zeta }}_{\text{BS}}=\frac{1}{N-M}\sum_{k=M+1}^{N}\zeta^{\left(k\right)},\\ {\hat{\zeta }}_{\text{BL}}=\frac{-1}{c}\log\left(\frac{1}{N-M}\sum_{k=M+1}^{N}e^{-c\zeta^{\left(k\right)}}\right),\;c\neq 0, \end{array}$$
where *M* is the burn-in period and *ζ*^(*k*)^=*θ*^(*k*)^, *λ*^(*k*)^, *r*^(*k*)^(*t*), *h*^(*k*)^(*t*), and CV^(*k*)^.
(8) To compute the CRIs of *ζ*, order {*ζ*^*M*+1^, *ζ*^*M*+2^,…, *ζ*^*N*^} as {*ζ*^[1]^, *ζ*^[2]^,…, *ζ*^[*N* − *M*]^}. Then, the (1 − *γ*)100% symmetric CRIs of *ζ* is
  (32) $$\begin{array}{c} \left[\zeta_{\left(N-M\right)\left(\gamma /2\right)},\zeta_{\left(\text{N}-\text{M}\right)\left(1-\gamma /2\right)}\right]. \end{array}$$

## 4. Simulation Study

Monte Carlo simulations were implemented utilizing 1000 AT2PC samples for each simulation to compare the estimators of parameters *θ* and *λ* as well as some lifetime parameters *r*(*t*), *h*(*t*), and CV discussed in the preceding sections. By using Mathematica ver. 12, all computations were conducted. We conducted a simulation study using different combinations of *T*, *n*, and *m* and different censored scheme **R**. We used the algorithm proposed by Ng et al. [6] to simulate an AT2PC sample from the PHFD with parameters (*θ*, *λ*)=(2,1.5). The true values of *r*(*t*), *h*(*t*), and CV at time *t*=0.4 are evaluated to be 0.9222, 0.506, and 0.4279, respectively. The performance of estimators is evaluated in terms of mean square error (MSE), which is computed as $\text{MSE}=1/N\sum_{k=1}^{N}{\left({\hat{\phi }}_{i}^{\left(k\right)}-\phi_{i}\right)}^{2}$, where *N*=10000, *i*=1,2,…, 5, *ϕ*_1_=*θ*, *ϕ*_2_=*λ*, *ϕ*_3_=*r*(*t*), *ϕ*_4_=*h*(*t*), and *ϕ*_5_=CV for the point estimates, also average lengths (ALs) and coverage probability (CPs), which are computed as the number of CIs that covered the true values divided by 1000, for interval estimates. Bayes estimates and the CRIs are computed based on (*N*=12000) MCMC samples and discard the first values (*M*=2000) as “burn-in.” In addition, we assume the informative gamma priors for *θ* and *λ*, that is, when the hyperparameters are *a*_*i*_=1 and *b*_*i*_=2, *i*=1,2. Moreover, 95% CRIs were computed for each simulated sample. In our study, we consider two different values of *T*=3, 5 and the following censoring schemes (CS):

- I: *R*_1_=*n* − *m*, *R*_*i*_=0 for *i* ≠ 1
- II: *R*_(*m*+1)/2_=*n* − *m*, *R*_*i*_=0 for *i* ≠ (*m*+1)/2 if *m* odd; *R*_*m*/2_=*n* − *m*, *R*_*i*_=0 for  *i* ≠ *m*/2 if *m* even
- III: *R*_*m*_=*n* − *m*, *R*_*i*_=0 for *i* ≠ *m*

The results of MSE, ALs, and CPs of estimates are shown in Tables 1–5.

## 5. Application of COVID-19 Data

In this section, for illustrative purposes, a real-life data for the coronavirus is presented to inspect the inference procedures discussed in the previous sections. We consider the set of real-life data, which is reported by Almongy et al. [26]. This data represents a COVID-19 mortality rate data that belongs to Italy of 59 days, which is recorded from 27 February to 27 April 2020. The data are as follows:

(33) $$\begin{array}{c} \begin{array}{cccccccccc} 4\text{.}571 & 7\text{.}201 & 3\text{.}606\; & 8\text{.}479 & 11\text{.}410 & 8\text{.}961 & 10\text{.}919 & 10\text{.}908 & 6\text{.}503 & 18\text{.}474\\ 11\text{.}010 & 17\text{.}337 & 16\text{.}561 & 13\text{.}226 & 15\text{.}137 & 8\text{.}697 & 15\text{.}787 & 13\text{.}333 & 11\text{.}822 & 14\text{.}242\\ 11\text{.}273 & 14\text{.}330 & 16\text{.}046 & 11\text{.}950 & 10\text{.}282 & 11\text{.}775 & 10\text{.}138 & 9\text{.}037 & 12\text{.}396 & 10\text{.}644\\ 8\text{.}646 & 8\text{.}905 & 8\text{.}906 & 7\text{.}407 & 7\text{.}445 & 7\text{.}214 & 6\text{.}194 & 4\text{.}640 & 5\text{.}452 & 5\text{.}073\\ 4\text{.}416 & 4\text{.}859 & 4\text{.}408 & \;4\text{.}639 & 3\text{.}148 & 4\text{.}040 & 4\text{.}253 & 4\text{.}011 & 3\text{.}564 & 3\text{.}827\\ 3\text{.}134 & 2\text{.}780 & 2\text{.}881 & 3\text{.}341 & 2\text{.}686 & 2\text{.}814 & 2\text{.}508 & 2\text{.}450 & 1\text{.}518 \end{array}. \end{array}$$

**Figure 4:**
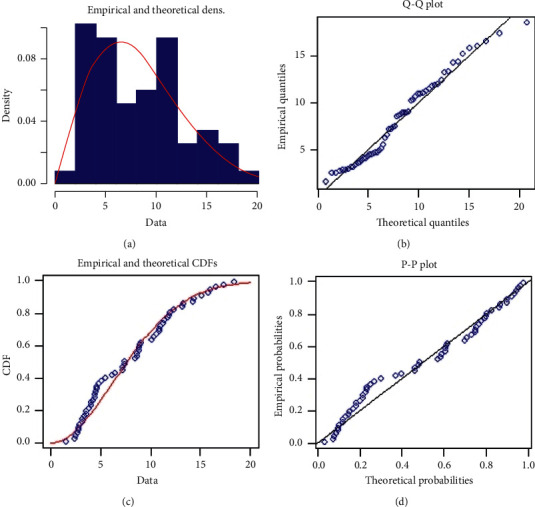
Graphical fitting of the PHFD.

For the goodness of fit test, we compute the Kolmogorov–Smirnov (K–S) distances between the empirical distribution and the fitted distribution functions. The K–S is 0.09254 and the associated *p*-value is 0.73124. Therefore, according to the *p*-value, we can say that the PHFD fits quite well to the above data. Empirically, *Q* − *Q* and *P* − *P* plots are shown in Figure 4, which clearly show that the PHFD fits the data very well.

Under the previous data, we use the algorithm proposed by Ng et al. [6] to generate an AT2PC sample with *m*=30, *T*=5, and *R*= {3, 0, 2, 0, 1, 0, 2, 0, 0, 3, 0, 3, 0, 1, 2, 0, 0, 3, 0, 0, 2, 1, 0, 3, 0, 1, 0, 2, 0, 0}. Thus, the resulting AT2PC sample is as follows:

(34) $$\begin{array}{c} \begin{array}{cccccccccc} 1\text{.}518 & 2\text{.}450 & 2\text{.}508 & 2\text{.}686 & 2\text{.}780 & 2\text{.}814 & 2\text{.}881 & 3\text{.}148 & 3\text{.}341 & 3\text{.}564\\ 3\text{.}827 & 4\text{.}040 & 4\text{.}253 & 4\text{.}408 & 4\text{.}416 & 4\text{.}571 & 5\text{.}073 & 5\text{.}452 & 7\text{.}201 & 7\text{.}214\\ 7\text{.}445 & 8\text{.}479 & 10\text{.}139 & 10\text{.}282 & 10\text{.}908 & 11\text{.}273 & 11\text{.}822 & 11\text{.}950 & 14\text{.}242 & 16\text{.}046 \end{array}. \end{array}$$

Based on the previous sample of AT2PC, the MLEs and ACIs for *θ*, *λ*, *r*(0.4), *h*(0.4), and *CV* are determined to be as in Table 6. Moreover, to compute the Bayesian estimates, the prior distributions of the parameters need to be specified. Since we have no prior information, we assume that noninformative gamma priors for *θ* and *λ*, that is, when the hyperparameters are *a*_*i*_=0.0001 and *b*_*i*_=0.0001, *i*=1,2. Under MCMC technique, the posterior analysis was done across combining M-H algorithm within Gibbs sampler. To conduct the MCMC algorithm which was described in Section 3.1, the initial values of the parameters *θ* and *λ* were taken to be their MLEs. In addition, 12000 MCMC samples were generated. To avoid the effect of the initial values (starting point), we expunge the first 2000 samples as “burn-in.” Table 6 shows the Bayesian estimates as well as 95% CRIs for *θ*, *λ*, *r*(0.4), *h*(0.4), and *CV*.

## 6. Conclusion

The purpose of this paper is to develop different methods to estimate the unknown quantities *θ*, *λ*, *r*(*t*), *h*(*t*), and CV of the PHFD using AT2PC scheme, which is introduced by Ng et al. [6]. The MLEs as well as ACIs using asymptotic distributions are obtained. Furthermore, to obtain the CIs of the reliability characteristics and coefficient of variation, we used delta method. It is clear that, after studying the Bayesian estimates, the posterior distribution equations of the unknown quantities are complicated and so hard to reduce analytically to well-known forms. For this reason, we have applied MCMC technique to compute the Bayes estimators. The Bayes estimates have been computed under both SE and LINEX loss functions. For illustrative purpose, real data set of COVID-19 mortality rates is considered. To check and compare the performance of the proposed methods, a simulation study was implemented with different sample sizes (*T*, *n*, *m*) and different CSs (I, II, III). According to the results, we note the following:

1. As expected, from Tables 1–5, as sample sizes (*n*, *m*) increases, the MSEs and ALs decrease.
2. From Tables 1–5, it is clear that the MSEs and ALs for all estimates at time *T*=3 are smaller than those at time *T*=5.
3. For fixed sample sizes and observed failures, the first scheme *I* is the best scheme in the sense of having smaller MSEs and ALs.
4. Bayes' estimates have the smallest MSEs and ALs for all estimates. Hence, Bayes' estimates perform better than the MLEs.
5. Bayes' estimate under LINEX with *c*=0.5 provides better estimates for all estimates because of having the smallest MSEs.
6. Bayes' estimates under LINEX for the choice *c*=0.5 perform better than their estimates for the choice *c*=−0.5 in the sense of having smaller MSEs.
7. Bayes' estimates under SE perform better than their estimates under LINEX with *c*=−0.5 in the sense of having smaller MSEs.
8. From Tables 1–5, we observe that as the time *T* increases, the MSEs and ALs associated with all estimates increase.
9. Both MLE and Bayesian methods have very close estimates and their ACIs have quite high CPs (around 0.95). Also, the Bayesian CRIs have the highest CPs.

## Figures and Tables

**Table 1:** MSE, ALs, and CPs of estimates for the parameter *θ*.

(*T*, *n*, *m*)	CS	MLE	Bayesian			MLE		Bayesian	
			SE	LINEX		ACIs		CRIs	
				*c*=−0.5	*c*=0.5	ALs	CPs	ALs	CPs
(3,30,20)	I	0.0785	0.0654	0.0663	0.0632	2.5355	0.921	2.1011	0.945
	II	0.0813	0.0764	0.0786	0.0746	2.6472	0.932	2.3125	0.938
	III	0.0856	0.0827	0.0835	0.0778	2.8141	0.926	2.5212	0.941
(3,50,30)	I	0.0723	0.0614	0.0625	0.0567	2.1457	0.934	1.7855	0.951
	II	0.0756	0.0661	0.0675	0.0594	2.3655	0.957	1.9567	0.961
	III	0.0786	0.0723	0.0736	0.0657	2.5471	0.935	2.0248	0.947
(3,75,50)	I	0.0663	0.0526	0.0539	0.0489	1.6899	0.948	1.4797	0.953
	II	0.0689	0.0578	0.0586	0.0525	1.7436	0.943	1.5963	0.954
	III	0.0711	0.0628	0.0634	0.0579	1.8654	0.949	1.6387	0.953
(5,30,20)	I	0.0896	0.0817	0.0824	0.0756	2.8992	0.938	2.5470	0.944
	II	0.0913	0.0863	0.0874	0.0799	2.9245	0.941	2.6894	0.948
	III	0.0934	0.0897	0.0905	0.0846	2.9652	0.949	2.7333	0.953
(5,50,30)	I	0.0846	0.0798	0.0803	0.0713	2.5634	0.941	1.9999	0.946
	II	0.0884	0.0865	0.0869	0.0754	2.6391	0.951	2.1368	0.963
	III	0.0929	0.0914	0.0921	0.0815	2.7452	0.949	2.4786	0.958
(5,75,50)	I	0.0735	0.0718	0.0726	0.0633	1.8947	0.951	1.5648	0.956
	II	0.0776	0.0752	0.0764	0.0685	1.9544	0.949	1.7865	0.974
	III	0.0819	0.0781	0.0796	0.0716	2.1345	0.947	1.9845	0.959

**Table 2:** MSE, ALs, and CPs of estimates for the parameter *λ*.

(*T*, *n*, *m*)	CS	MLE	Bayesian			MLE		Bayesian	
			SE	LINEX		ACIs		CRIs	
				*c*=−0.5	*c*=0.5	ALs	CPs	ALs	CPs
(3,30,20)	I	0.0456	0.0413	0.0426	0.0396	3.1453	0.939	2.7969	0.941
	II	0.0477	0.0438	0.0446	0.0416	3.3451	0.948	2.9455	0.958
	III	0.0489	0.0458	0.0466	0.0425	3.5660	0.936	3.1555	0.947
(3,50,30)	I	0.0412	0.0375	0.0382	0.0336	2.7994	0.947	2.4755	0.951
	II	0.0439	0.0399	0.0404	0.0361	2.9456	0.936	2.6363	0.948
	III	0.0458	0.0427	0.0436	0.0399	3.1887	0.929	2.8623	0.958
(3,75,50)	I	0.0355	0.0314	0.0325	0.0288	2.2365	0.936	1.9945	0.947
	II	0.0376	0.0356	0.0361	0.0329	2.4569	0.951	2.1479	0.956
	III	0.0407	0.0387	0.0396	0.0354	2.7695	0.958	2.3694	0.961
(5,30,20)	I	0.0661	0.0639	0.0648	0.0521	3.5891	0.934	3.1114	0.965
	II	0.0692	0.0665	0.0674	0.0567	3.7561	0.939	3.3654	0.947
	III	0.0747	0.0723	0.0738	0.0639	3.8956	0.941	3.5666	0.951
(5,50,30)	I	0.0559	0.0536	0.0544	0.0457	3.2548	0.918	2.9457	0.939
	II	0.0593	0.0576	0.0584	0.0483	3.4568	0.925	3.1452	0.947
	III	0.0665	0.0627	0.0637	0.0549	3.7655	0.928	3.4690	0.947
(5,75,50)	I	0.0422	0.0409	0.0399	0.0326	2.7966	0.938	2.2310	0.947
	II	0.0478	0.0453	0.0468	0.0356	2.9145	0.958	2.4777	0.961
	III	0.0511	0.0478	0.0489	0.0376	3.1321	0.949	2.5562	0.955

**Table 3:** MSE, ALs and CPs of estimates for *r*(*t*).

(*T*, *n*, *m*)	CS	MLE	Bayesian			MLE		Bayesian	
			SE	LINEX		ACIs		CRIs	
				*c*=−0.5	*c*=0.5	ALs	CPs	ALs	CPs
(3,30,20)	I	0.0256	0.0217	0.0223	0.0195	0.3546	0.942	0.2865	0.951
	II	0.0273	0.0246	0.0257	0.0219	0.3855	0.935	0.3159	0.947
	III	0.0315	0.0284	0.0296	0.0256	0.4163	0.932	0.3674	0.949
(3,50,30)	I	0.0212	0.0199	0.0209	0.0164	0.3144	0.925	0.2569	0.938
	II	0.0256	0.0228	0.0235	0.0189	0.3465	0.912	0.2932	0.941
	III	0.0291	0.0264	0.0276	0.0227	0.3774	0.925	0.3256	0.948
(3,75,50)	I	0.0156	0.0138	0.0149	0.0115	0.2761	0.939	0.2234	0.957
	II	0.0194	0.0166	0.0176	0.0141	0.2998	0.947	0.2611	0.966
	III	0.0235	0.0196	0.0211	0.0187	0.3266	0.939	0.2998	0.954
(5,30,20)	I	0.0331	0.0294	0.0315	0.0245	0.4154	0.939	0.3722	0.959
	II	0.0366	0.0348	0.0359	0.0291	0.4465	0.936	0.4100	0.947
	III	0.0412	0.0380	0.0398	0.0342	0.4867	0.928	0.4568	0.941
(5,50,30)	I	0.0273	0.0234	0.0246	0.0202	0.3567	0.954	0.2994	0.955
	II	0.0344	0.0317	0.0325	0.0269	0.3769	0.947	0.3325	0.961
	III	0.0395	0.0369	0.0378	0.0312	0.3966	0.939	0.3678	0.949
(5,75,50)	I	0.0213	0.0187	0.0199	0.0169	0.2999	0.941	0.2413	0.952
	II	0.0269	0.0245	0.0257	0.0188	0.3255	0.939	0.2864	0.957
	III	0.0298	0.0267	0.0279	0.0215	0.3710	0.940	0.3387	0.949

**Table 4:** MSE, ALs, and CPs of estimates for *h*(*t*).

(*T*, *n*, *m*)	CS	MLE	Bayesian			MLE		Bayesian	
			SE	LINEX		ACIs		CRIs	
				*c*=−0.5	*c*=0.5	ALs	CPs	ALs	CPs
(3,30,20)	I	0.0082	0.0077	0.0079	0.0072	0.6235	0.939	0.5494	0.946
	II	0.0085	0.0081	0.0082	0.0074	0.6641	0.950	0.5772	0.951
	III	0.0087	0.0084	0.0085	0.0077	0.6994	0.941	0.6156	0.948
(3,50,30)	I	0.0076	0.0072	0.0073	0.0066	0.5569	0.939	0.4756	0.955
	II	0.0078	0.0075	0.0076	0.0071	0.5836	0.954	0.5199	0.961
	III	0.0080	0.0077	0.0079	0.0074	0.6123	0.948	0.5644	0.953
(3,75,50)	I	0.0065	0.0062	0.0063	0.0058	0.4863	0.953	0.3956	0.948
	II	0.0069	0.0066	0.0067	0.0062	0.5236	0.946	0.4387	0.952
	III	0.0075	0.0072	0.0073	0.0067	0.5722	0.954	0.4867	0.956
(5,30,20)	I	0.0088	0.0082	0.0083	0.0077	0.7499	0.949	0.6722	0.961
	II	0.0091	0.0086	0.0087	0.0081	0.7935	0.960	0.7155	0.965
	III	0.0095	0.0091	0.0093	0.0085	0.8321	0.954	0.7601	0.949
(5,50,30)	I	0.0081	0.0077	0.0079	0.0071	0.6535	0.938	0.5767	0.945
	II	0.0085	0.0082	0.0083	0.0074	0.6944	0.951	0.6258	0.953
	III	0.0087	0.0085	0.0086	0.0079	0.7312	0.949	0.6699	0.956
(5,75,50)	I	0.0074	0.0069	0.0071	0.0065	0.5469	0.951	0.4777	0.961
	II	0.0079	0.0075	0.0077	0.0071	0.5861	0.954	0.5231	0.948
	III	0.0083	0.0079	0.0081	0.0076	0.6324	0.949	0.5697	0.952

**Table 5:** MSE, ALs, and CPs of estimates for CV.

(*T*, *n*, *m*)	CS	MLE	Bayesian			MLE		Bayesian	
			SE	LINEX		ACIs		CRIs	
				*c*=−0.5	*c*=0.5	ALs	CPs	ALs	CPs
(3,30,20)	I	0.0102	0.0092	0.0099	0.0087	1.0625	0.949	0.9298	0.951
	II	0.0135	0.0118	0.0127	0.0091	1.1542	0.939	0.9874	0.952
	III	0.0168	0.0143	0.00159	0.0098	1.2231	0.938	1.0561	0.948
(3,50,30)	I	0.0091	0.0087	0.0088	0.0075	0.9187	0.945	0.8255	0.954
	II	0.0096	0.0093	0.0094	0.0078	0.9763	0.952	0.8796	0.952
	III	0.0099	0.0096	0.0097	0.0082	1.1875	0.950	0.9555	0.961
(3,75,50)	I	0.0074	0.0071	0.0072	0.0067	0.8599	0.947	0.7834	0.948
	II	0.0079	0.0077	0.0078	0.0072	0.9475	0.939	0.8566	0.950
	III	0.0084	0.0081	0.0083	0.0075	1.0122	0.945	0.9323	0.947
(5,30,20)	I	0.0156	0.0132	0.0146	0.0099	1.3547	0.929	1.0984	0.939
	II	0.0191	0.0179	0.0185	0.0118	1.5891	0.951	1.2354	0.958
	III	0.0223	0.0200	0.0217	0.0157	1.6932	0.948	1.4199	0.958
(5,50,30)	I	0.0111	0.091	0.0099	0.0085	1.1547	0.939	0.9277	0.947
	II	0.0163	0.0137	0.0148	0.0093	1.2369	0.945	1.1352	0.961
	III	0.0199	0.0175	0.0186	0.0103	1.4251	0.952	1.2874	0.948
(5,75,50)	I	0.0086	0.0083	0.0084	0.0077	0.9584	0.949	0.8756	0.951
	II	0.0091	0.0088	0.0089	0.0081	0.9999	0.952	0.9274	0.962
	III	0.0097	0.0094	0.0096	0.0085	1.1023	0.948	0.9876	0.955

**Table 6:** Point estimates, 95% ACIs and 95% CRIs of *θ*, *λ*, *r*(*t*), *h*(*t*), and *CV*.

Parameter	MLE	Bayesian			MLE	Bayesian
		SE	LINEX		ACIs	CRIs
			*c*=−0.5	*c*=0.5	[Lower, upper]	[Lower, upper]
*θ*	0.0360	0.0315	0.0342	0.0299	[0.0115, 0.0606]	[0.0210, 0.0657]
*λ*	0.2494	0.2265	0.2337	0.2116	[0.0000, 0.5979]	[0.0112, 0.4799]
*r*(0.4)	0.9909	0.9800	0.9812	0.9795	[0.9797, 1.0021]	[0.9694, 1.0002]
*h*(0.4)	0.0287	0.0267	0.0276	0.0249	[0.0009, 0.0721]	[0.0084, 0.0583]
CV	0.8054	0.7587	0.7699	0.7501	[0.5924, 1.0184]	[0.4885, 0.9945]

## Data Availability

The datasets generated during the current study are available from the corresponding author on reasonable request.
